# Facemasks, Hand Hygiene, and Influenza among Young Adults: A Randomized Intervention Trial

**DOI:** 10.1371/journal.pone.0029744

**Published:** 2012-01-25

**Authors:** Allison E. Aiello, Vanessa Perez, Rebecca M. Coulborn, Brian M. Davis, Monica Uddin, Arnold S. Monto

**Affiliations:** 1 Department of Epidemiology, University of Michigan-School of Public Health, Ann Arbor, Michigan, United States of America; 2 Center for Social Epidemiology & Population, University of Michigan-School of Public Health, Ann Arbor, Michigan, United States of America; Fred Hutchinson Cancer Research Center, United States of America

## Abstract

Limited vaccine availability and the potential for resistance to antiviral medications have led to calls for establishing the efficacy of non-pharmaceutical measures for mitigating pandemic influenza. Our objective was to examine if the use of face masks and hand hygiene reduced rates of influenza-like illness (ILI) and laboratory-confirmed influenza in the natural setting. A cluster-randomized intervention trial was designed involving 1,178 young adults living in 37 residence houses in 5 university residence halls during the 2007–2008 influenza season. Participants were assigned to face mask and hand hygiene, face mask only, or control group during the study. Discrete-time survival models using generalized estimating equations to estimate intervention effects on ILI and confirmed influenza A/B infection over a 6-week study period were examined. A significant reduction in the rate of ILI was observed in weeks 3 through 6 of the study, with a maximum reduction of 75% during the final study week (rate ratio [RR] = 0.25, [95% CI, 0.07 to 0.87]). Both intervention groups compared to the control showed cumulative reductions in rates of influenza over the study period, although results did not reach statistical significance. Generalizability limited to similar settings and age groups. Face masks and hand hygiene combined may reduce the rate of ILI and confirmed influenza in community settings. These non-pharmaceutical measures should be recommended in crowded settings at the start of an influenza pandemic.

**Trail Registration:**

Clinicaltrials.gov NCT00490633

## Introduction

As part of planning for pandemic influenza, serious attention has been given to non-pharmaceutical interventions (NPIs) for prevention. This is based in part on the realization that vaccines and antiviral medications may be in short supply or unavailable at the start of a pandemic. A number of studies have recently been conducted to strengthen the scientific basis for recommendations on the use of specific influenza interventions [1]. These studies have primarily been performed during seasonal influenza outbreaks, but have recently been validated during the swine origin pandemic of 2009, in which the need for NPIs was reaffirmed. NPIs implemented at the beginning of the 2009 pandemic included home quarantine, isolation of the ill, social distancing, and personal protection measures (e.g. face masks and hand hygiene).

We began to examine the impact of NPIs on the incidence of seasonal influenza in 2006–2007, a year of modest influenza activity [2]. Our work demonstrated that the use of face masks and hand hygiene, combined, conferred protection from primary influenza-like illness (ILI) among young adults living in university residence halls [2]. We continued our research into the 2007–2008 influenza season with improvements in our study design aimed at a more efficient examination of intervention effects on rates of ILI and laboratory-confirmed influenza. In contrast to our earlier study, the 2007–2008 season was characterized by higher levels of influenza activity. In this paper, we present findings from the 2007–2008 season of our cluster-randomized intervention trial of face masks and hand hygiene for preventing ILI and laboratory-confirmed influenza.

## Methods

The protocol and supporting CONSORT checklist are available as supporting information; see Checklist S1 and Protocol S1.

### Ethics statement

The study was approved by the Institutional Review Board at the University of Michigan, HUM00008566. Informed consent was obtained by all participants through electronic signature of the online form, as approved by the Institutional Review Board.

### Study design

A cluster-randomized intervention trial (Mflu) was conducted at the University of Michigan (trial registration: Intervention Study of Face Mask and Hand Sanitizer to Reduce Influenza Transmission (M-FLU), Identifier: NCT00490633, trial link: http://www.clinicaltrials.gov/ct2/show/NCT00490633). Findings from the first year of Mflu for the 2006–2007 flu season have been published [2]. The 2007–2008 trial described here followed 1,178 young adults living within university residence halls during the influenza season and included a significantly larger number of clusters for randomization. Thirty-seven residence houses located in five residence halls were randomly assigned to either an intervention or a control group. Students living in these residence halls were eligible for the study if they were at least 18 years of age, willing to wear a face mask, use alcohol-based hand sanitizer, provide a throat swab specimen when sick, and complete one baseline and six weekly on-line surveys. Students reporting an allergy to alcohol-based hand sanitizer were excluded. Based on data from year one of the Mflu study [2] and assuming an 8% observable ILI attack rate in the control group, we had 87% power to detect a reduction of 25% (i.e. a rate ratio [RR] = 0.75) or greater in illness rates between intervention and control groups at an α-level 0.05, using the methods of Hayes et al for cluster randomized trials [3]. The CONSORT checklist is presented in Checklist S1.

### Randomization and intervention

Randomization at the residence house level was performed using Proc Plan (SAS v. 9.1 Cary, NC) by study staff (see Text S1 section I for further details on randomization). A residence house is defined as a shared “hall way”, “wing”, or “floor (s)” containing several dorm rooms that share common areas or bathrooms. For participating residence halls, the average number of residence houses in each was 7.4 (range: 4 to 9). All residence houses in each of the residence halls were randomized prior to the intervention implementation. Recruitment by study staff began in November 2007 and continued through February 2008. The intervention period began during the week of January 28, 2008 following laboratory-confirmation of influenza on campus through ongoing surveillance at the University Health Services. Intervention materials and a required educational video on proper hand hygiene and use of standard medical procedure face masks were provided to study participants on January 24^th^. There was also a one-week spring break during the study when a majority of students left campus (February 23^rd^–March 2^nd^) and therefore illness symptoms that may have occurred during this period were not assessed. Excluding the break, interventions were implemented for 6 weeks (i.e. 42 days) and ended on March 14^th^.

The intervention groups included mask and hand hygiene or mask alone. Participants in the face mask and hand hygiene and the face mask only groups received weekly packets of mask supplies in their student mailboxes. Each packet included seven standard medical procedure masks with ear loops (TECNOL™ procedure masks, Kimberly-Clark, Roswell GA) and plastic bags for storage during interruptions in mask use (e.g., while eating, sleeping, etc.) and for daily disposal. Participants were asked to wear their masks for at least six hours per day while in their residence hall. Students were encouraged but not obligated to wear their face masks outside of their residence hall. In addition to masks, all participants in the face mask and hand hygiene intervention received hand sanitizer (2 oz squeeze bottle, 8 oz pump bottle with 62% ethyl alcohol in a gel base). The control group did not receive an intervention. Additional information on supply distribution is presented in (Text S1 section I).

### Weekly surveys

Participants were asked to provide self-reported data at baseline on demographic information, hand hygiene practices, health behaviors, smoking habits, vaccination, and perceived stress [4]. Participants were also asked to complete on-line weekly surveys and to report the presence/absence of illness symptoms. Weekly surveys included questions on ILI symptoms, intervention compliance (e.g. total mask hours per day and frequency of alcohol-based hand sanitizer use), and health and hand hygiene practices. Detailed descriptions of additional behavioral and compliance measures are presented online in Text S1 (see sections II and III).

### ILI symptoms and laboratory testing

All study participants were given materials describing the ILI case definition (presence of cough and at least one or more of fever/feverishness, chills, or body aches) and contact information of clinical research staff for illness assessment. Clinical research staff recorded the date of illness onset, body temperature, use of anti-pyretics, and reported symptoms. Throat swab specimens were tested for influenza A or B using real-time polymerase chain reaction (Rt-PCR). Positive samples were identified using PCR samples tested using the TaqMan System (Applied Biosystem, Foster City, CA, USA). Primers and probes were developed by the CDC Influenza Branch to detect influenza types A and B as previously noted [5]. Information on laboratory procedures are included online in Text S1 section IV.

### Statistical analysis

The objective of this study was to assess whether the application of masks or masks and hand hygiene together among a generally health student population reduces influenza and ILI compared to a control group not receiving these interventions. We hypothesized that there would be a significant reduction in influenza and ILI in both the mask and hand hygiene and mask alone groups compared to the control group. This was a single blind study where the PI's and statisticians were blinded to intervention status during analyses. Imbalances in baseline study characteristics were examined between intervention and control groups using cluster-adjusted chi-squared tests and cluster-adjusted ANOVA [6]. Intracluster correlation coefficients (ICCs) and corresponding *P* values were calculated in R software using the Donner method [7], [8].

#### Compliance

Compliance measures were log transformed to account for skewness. Values of 0 were given a value of 1 prior to log transformation. Differences in compliance between intervention and control groups were examined using multi-level mixed models in SAS Proc Mixed (SAS V.9.1, Cary, NC). Level-1 accounted for changes between individuals in compliance over the course of the study period. Level-2 allowed for changes in compliance within individuals across the study period. Random intercepts were used to account for clustering by residence house. The type III F-test and corresponding *P* value were computed for an overall comparison in compliance between the intervention and control groups. Weekly comparisons between groups were also evaluated. *P* values were set to ≤0.025 for statistical significance to account for multiple comparisons in week-by-week analyses.

#### Intervention

**Figure 1:**
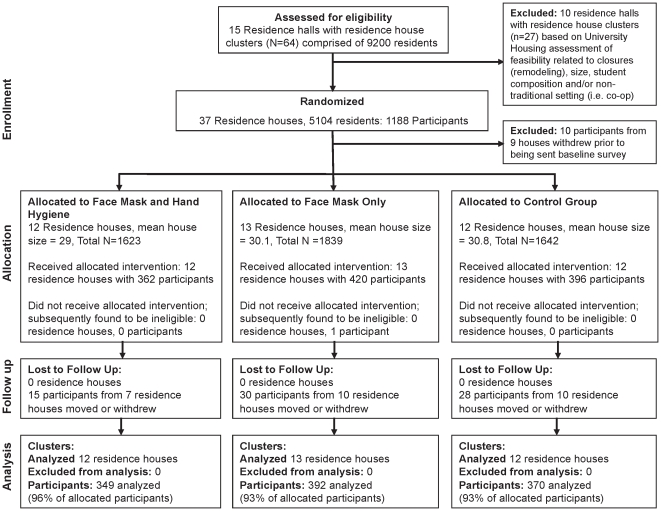
Flow chart of participants throughout the study period. This figure shows the enrollment, allocation, follow-up, and analysis numbers for the study.

The main predictor variable was an indicator for whether the individual was in an intervention group (mask and hand hygiene or mask alone) or control group (as the referent). The main outcome variables measured at the individual level were time to first ILI and time to PCR-confirmed influenza A/B during the study period. In total, 1,178 of 1,188 recruited students living in participating residence halls were eligible for study inclusion (see Figure 1). Of the 1,178 participants, 1,111 were available for statistical analyses (see Figure 1). For incident ILI, an ILI-free study population at baseline was examined (N = 938/1,111). For influenza, all participants (N = 1,111) were examined since there were no laboratory confirmed cases of influenza at baseline.

An intention-to-treat analysis was performed [9]. Log-survival and log-log survival plots showed that the proportional hazards assumption was not met. Therefore, discrete-time survival analysis was performed to examine intervention effects on time to first ILI or influenza infection [10]. Proc Genmod in SAS (SAS V.9.1, Cary, NC) was used to estimate rate ratios (RRs) and 95% confidence intervals (CIs) for each study week and cumulatively over the study period for ILI. Only cumulative RRs were examined for influenza since the number of cases was too small to generate weekly estimates. Generalized estimating equations were used to control for clustering [10], [11]. Variables measured at baseline were added to the final model if the magnitude of effect was more than a 10% increase or decrease from the null value (RR = 1.00) in univariate analyses. RRs were considered statistically significant at *P*<0.05.

## Results

### Demographics

A total of 1,111 eligible participants (94% retention) were available for analysis with 349 in face mask and hand hygiene, 392 in mask only, and 370 in the control group (see Figure 1). Baseline characteristics of participants are shown in Table S1. The mean age was 18.95 years (SD, 0.9). There were no statistically significant differences between study groups among any of the covariates examined (see Table S1).

### Compliance

**Figure 2:**
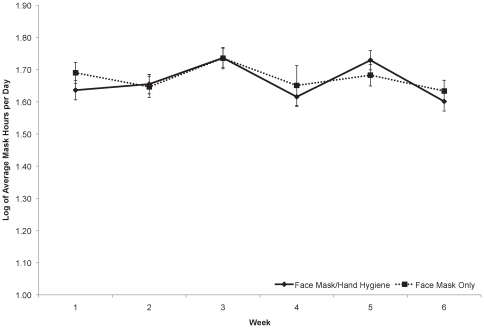
Reported daily average number of hours (log transformed) of facemask use by study week. This figure shows the daily average number of hours (log transformed) of facemask use by study week in both the face mask and hand hygiene group (solid line) and the face mask only group (dotted line). The type III fixed effects model for assessing differences over time using a week * group interaction term, was not statistically significant, F(5, 2943) = 1.30, *P* = 0.26.

Compliance analyses demonstrated that subjects in the face mask and hand hygiene group wore their mask, on average, 5.08 hours per day (SD, 2.23) compared to subjects in the mask only group (5.04 hours per day [SD, 2.20]) (see Figure 2). No significant difference in mask use between the two interventions was observed throughout the study (see Figure 2).

Alcohol-based hand sanitizer use (study provided or personally owned) was examined among subjects in each of the three study groups. The face mask and hand hygiene group reported an average use of hand sanitizer 4.49 times per day (SD, 4.10). The mask only group reported an average use of hand sanitizer 1.29 times per day (SD, 1.77) and the control group reported use of 1.51 times per day (SD, 2.25). The face mask and hand hygiene group used hand sanitizer significantly more often compared to subjects in either the mask only or control groups (see Table 1). No significant differences were observed between the mask only and control group. Results for additional compliance measures are described in Text S1 section III and shown in tables S2, S3, S4, S5, S6, S7 and figures S1, S2, S3, S4.

**Table 1:** Reported average (log-transformed) daily alcohol-based hand sanitizer use per week.

Log reported average daily alcohol based hand sanitizer use per week and *P* values comparing average use in each group with face mask and hand hygiene							
Intervention	Average use over all weeks^a^	Week 1	Week 2	Week 3	Week 4	Week 5	Week 6
Face Mask and Hand Hygiene	1.49	1.47	1.48	1.53	1.37	1.40	1.40
vs. Face Mask Only^b^	0.61	0.65	0.61	0.62	0.58	0.57	0.60
		(*P*<.001)	(*P*<.001)	(*P*<.001)	(*P*<.001)	(*P*<.001)	(*P*<.001)
vs. Control	0.66	0.70	0.70	0.69	0.59	0.62	0.65
		(*P*<.001)	(*P*<.001)	(*P*<.001)	(*P*<.001)	(*P*<.001)	(*P*<.001)

a The change in reported average log transformed daily alcohol-based hand sanitizer use over the 6 week period comparing between all three study groups (week*group interaction term) using a Type III fixed effects model resulted in an *F*(10, 4431) = 1.18 and *P* = 0.30.

b There were no statistically significant differences at any weeks comparing hand sanitizer use between face mask only and the control group (all *P*>0.025).

### ILI and laboratory-confirmed influenza

One hundred and seventy three of 1,111 participants reported ILI on their baseline survey. The remaining 938 subjects were considered ILI-free and analyzed for incident ILI during the study period. Factors associated with incident ILI included gender, race, ethnicity, smoking, physical activity, and having ever received an influenza vaccination (see Table 2). The proportion of subjects with ILI and laboratory-confirmed influenza by study group are shown in Table S5. Of the 938 ILI-free participants at baseline, 128 (14%) subsequently met the case definition of ILI throughout the study period. Of these 128 ILI cases, 34 subjects tested positive by Rt-PCR for influenza infection (27%).

**Table 2:** Unadjusted associations between demographic characteristics and rate of influenza-like illness among subjects who were ILI free at baseline (N = 938).

Characteristic	ICC^a^	RR	95% CI	*P* b
Age at baseline	0.0019	1.06	(0.87–1.28)	0.58
Gender female vs. male	0.0014	1.61	(1.11–2.33)	0.01
Race (ref White)	0.0014			
black		0.28	(0.09–0.87)	0.03
Asian		0.90	(0.57–1.41)	0.63
Other		0.56	(0.22–1.37)	0.20
Ethnicity				
Hispanic or Latino vs. not Hispanic or Latino	0.0018	1.69	(0.88–3.24)	0.11
Sleep quality good vs. bad	0.0017	1.01	(0.65–1.56)	0.98
Stress score	0.0017	0.99	(0.97–1.01)	0.33
Smoking current vs. non	0.0011	2.39	(1.04–5.49)	0.04
Alcohol consumption (ref 0 drinks/week)	0.0013			
1 or more drinks		1.03	(0.70–1.50)	0.90
Physical activity high vs. low	0.0013	1.30	(0.90–1.89)	0.16
Flu shot ever vs. never	0.0017	1.27	(0.88–1.81)	0.20
Recent shot yes vs. no	0.0019	0.95	(0.59–1.52)	0.82
Optimal handwashing at baseline yes vs. no	0.0019	0.88	(0.58–1.34)	0.56
Hand sanitizer ownership yes vs. no	0.0017	1.00	(0.70–1.42)	0.99

a ICC = Intracluster correlation coefficient; RR = rate ratio; 95% CI = 95% confidence interval.

b Variables added to the final adjusted model if the magnitude of effect was more than a 10% increase or decrease from the null value (RR = 1.00).

At week 3 and onward, significantly reduced ILI rates were observed in the face mask and hand hygiene group compared to the control in adjusted models (see Table 3). The largest reduction was observed during week 6 with a 75% reduced ILI rate (adjusted RR = 0.25, [95% CI, 0.07 to 0.87]) among subjects in the face mask and hand hygiene group in adjusted models. Statistically significant findings were not observed for the face mask only group when compared to the control group (see Table 3).

**Table 3:** Intervention rate ratios for influenza-like illness.

	Unadjusted Model a					
	Face Mask vs. Control			Face Mask/Hand hygiene vs. Control		
Week	RR^b^	95% CI^c^	*P*	RR^b^	95% CI^c^	*P*
1	0.80	(0.41–1.53)	0.49	0.99	(0.51–1.93)	0.98
2	0.86	(0.52–1.40)	0.53	0.78	(0.47–1.29)	0.33
3	0.92	(0.62–1.37)	0.68	0.61	(0.37–1.01)	0.06
4	0.99	(0.64–1.52)	0.96	0.48	(0.24–0.94)	0.03^e^
5	1.06	(0.61–1.87)	0.83	0.38	(0.15–0.94)	0.04^e^
6	1.14	(0.54–2.41)	0.72	0.30	(0.09–0.98)	0.05
**Cumulative Rate Ratio** d	1.08	(0.86–1.34)	0.52	0.78	(0.59–1.05)	0.10

	Adjusted Model a					
	Face Mask vs. Control			Face Mask/Hand hygiene vs. Control		
1	0.64	(0.34–1.19)	0.16	0.85	(0.44–1.64)	0.62
2	0.70	(0.44–1.14)	0.15	0.66	(0.40–1.10)	0.11
3	0.77	(0.51–1.17)	0.23	0.52	(0.30–0.88)	0.02^e^
4	0.85	(0.53–1.36)	0.49	0.40	(0.20–0.83)	0.01^e^
5	0.93	(0.51–1.71)	0.82	0.32	(0.12–0.84)	0.02^e^
6	1.02	(0.46–2.25)	0.96	0.25	(0.07–0.87)	0.03^e^
**Cumulative Rate Ratio** d	1.10	(0.88–1.38)	0.42	0.78	(0.57–1.08)	0.13

a Intracluster correlation coefficient: 0.0004 in unadjusted model (N = 938), −0.0005 in model adjusting for gender, race, ethnicity, smoking status, physical activity, and having ever received a vaccination for influenza (N = 828).

b RR, rate ratio.

c 95% CI, 95% confidence interval.

d Cumulative rate ratio is the week by treatment effect which is equivalent to the hazard ratio over the study period.

e Significance level set at *P*<0.05.

Table 4 shows the cumulative RRs for laboratory-confirmed influenza. The face mask and hand hygiene group and the face mask only group compared to the control showed a 43% (adjusted RR = 0.57, [CI, 0.26 to 1.24]) and 8% (adjusted RR = 0.92, [CI, 0.59 to 1.42]) reduction in the cumulative rate of influenza, respectively, throughout the study.

**Table 4:** Intervention rate ratios for influenza infection.

Unadjusted Model a					
Face Mask vs. Control			Face Mask/Hand hygiene vs. Control		
cRR^b^	95% CI^c^	*P* Value	RR^b^	95% CI^c^	*P* Value
0.93	(0.60–1.42)	0.72	0.57	(0.26–1.24)	0.15

Adjusted Model a					
0.92	(0.59–1.42)	0.69	0.57	(0.26–1.24)	0.16

a Intracluster correlation coefficient: −0.0014 in unadjusted model (N = 1,111), −0.0030 in model adjusting for gender, race, ethnicity, smoking status, physical activity, and having ever received a vaccination for influenza (N = 986).

b cRR = Cumulative Rate Ratio is the week by treatment effect which is equivalent to the hazard ratio over the study period.

c 95% CI, 95% confidence interval. Significance level set at *P*<0.05.

## Discussion

We examined the efficacy of face masks and hand hygiene for reducing the incidence of ILI and laboratory-confirmed influenza in an open, non-institutionalized population of young adults. Our findings show a significant reduction in the rate of ILI among participants randomized to the face mask and hand hygiene intervention during the latter half of the study period, ranging from 48% to 75% when compared to the control group. We also observed a substantial (43%) reduction in the incidence of influenza infection in the face mask and hand hygiene group compared to the control, but this estimate was not statistically significant. There were no substantial reductions in ILI or laboratory-confirmed influenza in the face mask only group compared to the control. Our ILI findings are consistent with results from the first year of this two-year study [2] and a previous literature review on studies examining the efficacy of mask use in reducing transmission of respiratory viruses [12]. There are no other mask and hand hygiene intervention studies, to our knowledge, that have examined if wearing a mask prior to illness and jointly practicing hygiene prevents illness for the person practicing the intervention. The majority of earlier studies examined the impact of wearing a mask after a household member had been identified as an ILI or influenza case [13], [14], [15], [16]. Our study, therefore, is an important contribution to understanding the effectiveness of these interventions for mitigating influenza outbreaks and possibly pandemic scenarios in crowded and close living environments before outbreaks ensue.

Although few data are available to evaluate the efficacy of mask use in the community setting [17], four recent randomized mask intervention trials examined the impact of mask use on secondary transmission of ILI, upper respiratory infection and/or influenza in households [13], [14], [15], [16]. Cowling et al. [13] showed a reduction of 67% in influenza infection when masks were donned within 36 hours of the index case's symptom onset. Canini et al. [16] found no association between intervention households providing the primary case with masks compared to control households with no masks; the authors did, however, report a severely underpowered study due to early termination of the intervention. MacIntyre et al. [14] showed a borderline significant reduction in ILI among study participants using P2 masks but only 21% complied with mask use. Larson et al. [15] found no significant difference between targeted education, education with use of hand sanitizer, and education with masks and hand hygiene for overall rates of upper respiratory infection (URI); but, face masks were associated with a reduced secondary attack rate [15]. In contrast to these earlier studies, our design allowed us to follow disease-free participants at baseline who were asked to wear masks and/or conduct hygiene for the entire follow-up period, not just when they or their contacts were ill, thus limiting the potential for infection prior to mask adoption. Furthermore, our study design more accurately represents guidelines and plans that call for use of NPIs before susceptible individuals become ill [18].

In both this study and in our earlier work [2] we identified significant reductions in ILI rates several weeks into the study. Increases in compliance with hand hygiene measures may partly explain why we observed a significant reduction during the latter half of the study period over two different influenza seasons with different participants and cluster sample sizes [2]. First, a significantly higher proportion of those in the face mask and hand hygiene group reported using at least a quarter size or greater amount of hand sanitizer (equivalent to at least one full pump from an 8 oz. bottle) compared to both the face mask only and control groups starting at week 4 in this study (see Text S1 section III). Hence, greater adherence with hand sanitizer use throughout the study period and a significant difference in the amount used in the latter half of this period may have contributed to the increased reductions in ILI rates observed during the latter half of the study period. Next, in both our 2006–07 and 2007–08 studies, we increased notifications regarding mask compliance to subjects in either mask intervention group and hand hygiene compliance to subjects in the face mask and hand hygiene group when they returned from spring break. Spring break occurred before the fourth week of the study and this may have led to improved compliance with hand hygiene and mask hours during the latter half of the period when compliance messaging was enhanced. Although we did not identify a significant increase in mask compliance during this time, it is possible that the participants became more comfortable with proper use of the masks as the study progressed. We were able to collect survey information over the study period on mask comfort and found that there was a slight increase in reported comfort for the mask groups beginning at week four (see Text S1 section III).

Our RRs for comparing the face mask and hand hygiene group to the control group suggest an overall reduction in the primary incidence of influenza. However, we only analyzed 34 incident cases of influenza during the study and this limited our statistical power. Nonetheless, the RR for the layered intervention group was of even greater magnitude and in the same direction as the cumulative RR for ILI. These trends suggest that face masks and hand hygiene should be encouraged during seasonal influenza outbreaks and especially during the beginning of a pandemic when vaccines may not yet be available.

This research has several limitations. First, it is possible that participants with ILI who tested negative for influenza were infected with respiratory viruses other than influenza. Variations in ILI case definitions in surveillance studies contribute to the complexity of this issue. However, as supported by the literature [19], symptoms of cough and fever/feverishness were the two strongest predictors of confirmed influenza in our study and two symptoms constituting our ILI case definition, making it a good measure for influenza infection. Moreover, the attack rates for ILI peaked at the same time as our laboratory confirmed influenza cases (a subset of all ILI cases), suggesting that our ILI outcome followed a similar attack rates as laboratory confirmed influenza (see figure S5). Therefore, ILI cases without lab confirmed flu positivity may still have been flu cases that we were unable to detect in the lab. Since participants were only required to wear masks while in their residence hall, it is possible that transmission of infection occurred outside of the residential environment when masks were not in use. Nonetheless, students at the University of Michigan live, eat, study, and some can even take their classes within residence halls, suggesting that transmission is likely to be high in this crowded and interactive setting. In addition, we did not have the funds to include a hand hygiene only group and therefore cannot disentangle the combined effects of masks and hand hygiene. Additional limitations include our reliance on self-reported data, which may be susceptible to reporting and recall bias [20]. However, we used randomized assignment of interventions and found similarity in reported behavioral habits and hand hygiene practices across intervention and control groups at baseline, which argues against differential reporting biases. Generalizability of our study findings are limited to similar environmental settings and populations. Due to the inability to blind participants to study interventions, compliance with these interventions must be considered carefully. We observed compliance, but it was not possible to gather observational data on all participants at all times and venues.

Our study demonstrated a significant association between the combined use of face masks and hand hygiene and a substantially reduced incidence of ILI during a seasonal influenza outbreak. If masks and hand hygiene have similar impacts on primary incidence of infection with other seasonal and pandemic strains, particularly in crowded, community settings, then transmission of viruses between persons may be significantly decreased by these interventions. Masks alone did not provide a benefit, suggesting that single personal protective interventions do not protect against incidence of ILI or influenza. However, it is possible that either lack of power to detect small effects from mask use alone or that the amount of time masks were worn was not sufficient alone to provide a reduction in illness. Our timely findings regarding the efficacy of masks and hand hygiene highlight the significance of examining their impact on influenza infection within community settings.

## Supporting Information

Figure S1 **Reported daily average number of hand washes (log transformed) by study week.** This figure shows the daily average number of hand washes (log transformed) by study week in the face mask and hand hygiene group (solid line), the face mask only group (dotted line), and the control group (dashed line). The type III fixed effects model for assessing differences over time using a week * group interaction term, was not statistically significant, F(10, 4543) = 1.43 and P = 0.16.
(TIF)

Figure S2 **Reported daily average seconds of hand washing (log transformed) by study week.** This figure shows the daily average time for washing hands (log transformed) by study week in the face mask and hand hygiene group (solid line), the face mask only group (dotted line), and the control group (dashed line). The type III fixed effects model for assessing differences over time using a week * group interaction term, was not statistically significant, F(10, 4518) = 1.12 and P = 0.34.
(TIF)

Figure S3 **Reported daily average mask comfort rating (log transformed) by study week.** This figure shows the daily average mask comfort rating (log transformed) by study week in both the face mask and hand hygiene group (solid line) and the face mask only group (dotted line). The type III fixed effects model for assessing differences over time using a week * group interaction term, was not statistically significant, F(5, 2942) = 0.68 and P = 0.63.
(TIF)

Figure S4 **Reported average proportion of proper hand sanitizer use by study week.** This figure shows the average proportion of respondents using the proper amount of hand sanitizer (quarter size or larger) when using hand sanitizer by study week in the face mask and hand hygiene group (black), the face mask only group (dark grey), and the control group (light grey).
(TIF)

Figure S5 **Attack rate of influenza like-illness at the University of Michigan during the 2007–2008 influenza season.** The attack rate of influenza and influenza like-illness among respondents and across campus.
(TIF)

Table S1 **Baseline characteristics of the study population.**
(DOC)

Table S2 **Log reported average daily hand washing per week and P values comparing average washing in each group with face mask and hand hygiene.**
(DOC)

Table S3 **Log reported average wash time in seconds per week and** ***P*** **values comparing wash time in each group with face mask and hand hygiene.**
(DOC)

Table S4 **Log reported average face mask comfort per week and** ***P*** **values comparing comfort in the face mask only group with face mask and hand hygiene.**
(DOC)

Table S5 **Proportion of influenza-like illness cases who tested positive for influenza infection as determined by polymerase chain reaction (PCR).**
(DOC)

Table S6 **Proportion of subjects using a quarter or greater amount of alcohol sanitizer and** ***P*** **values comparing each group using the Donner and Donald chi-square test.**
(DOC)

Table S7 **Observational data based on the total hours of observation in each residence hall and the percentage of shifts in which participants were seen properly wearing facemasks.**
(DOC)

Text S1 **Additional methods and compliance measures.** This text provides additional information about randomization and distribution of supplies, behavioral measures collected, additional compliance measures, laboratory methods and study attack rate.
(DOC)

Checklist S1 **CONSORT checklist.** This text provides the clustered CONSORT checklist for our study.
(DOC)

Protocol S1 **Trial protocol.** This text provides additional information about the protocol of the study.
(PDF)
