# Supplementary figures and images for: Facemasks, Hand Hygiene, and Influenza among Young Adults: A Randomized Intervention Trial
Source: PLoS One. 2012 Jan 25;7(1):e29744. doi: 10.1371/journal.pone.0029744 (PMC3266257; doi:10.1371/journal.pone.0029744)

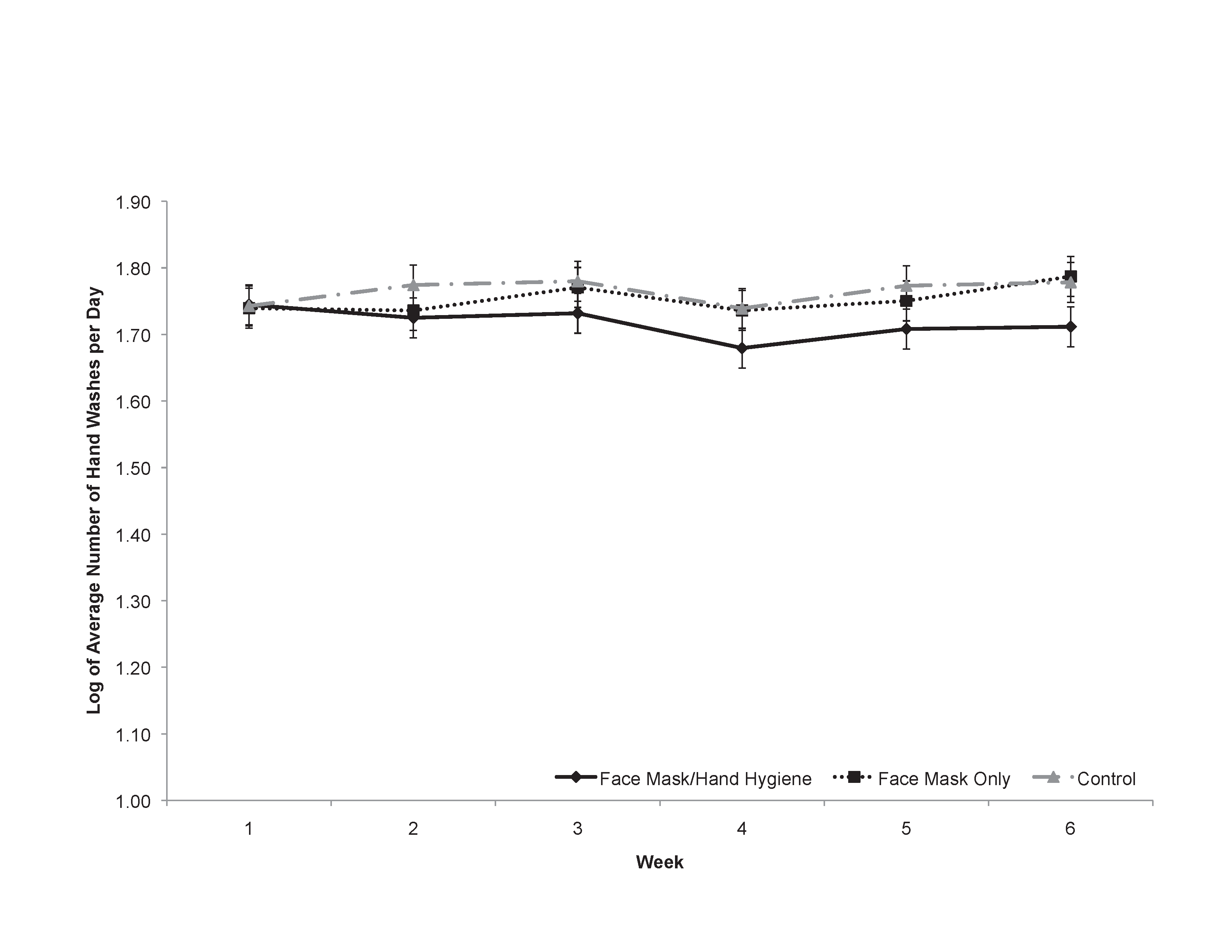

Supplement: Figure S1 — Reported daily average number of hand washes (log transformed) by study week. This figure shows the daily average number of hand washes (log transformed) by study week in the face mask and hand hygiene group (solid line), the face mask only group (dotted line), and the control group (dashed line). The type III fixed effects model for assessing differences over time using a week * group interaction term, was not statistically significant, F(10, 4543) = 1.43 and P = 0.16. (TIF) [file pone.0029744.s001.tif]

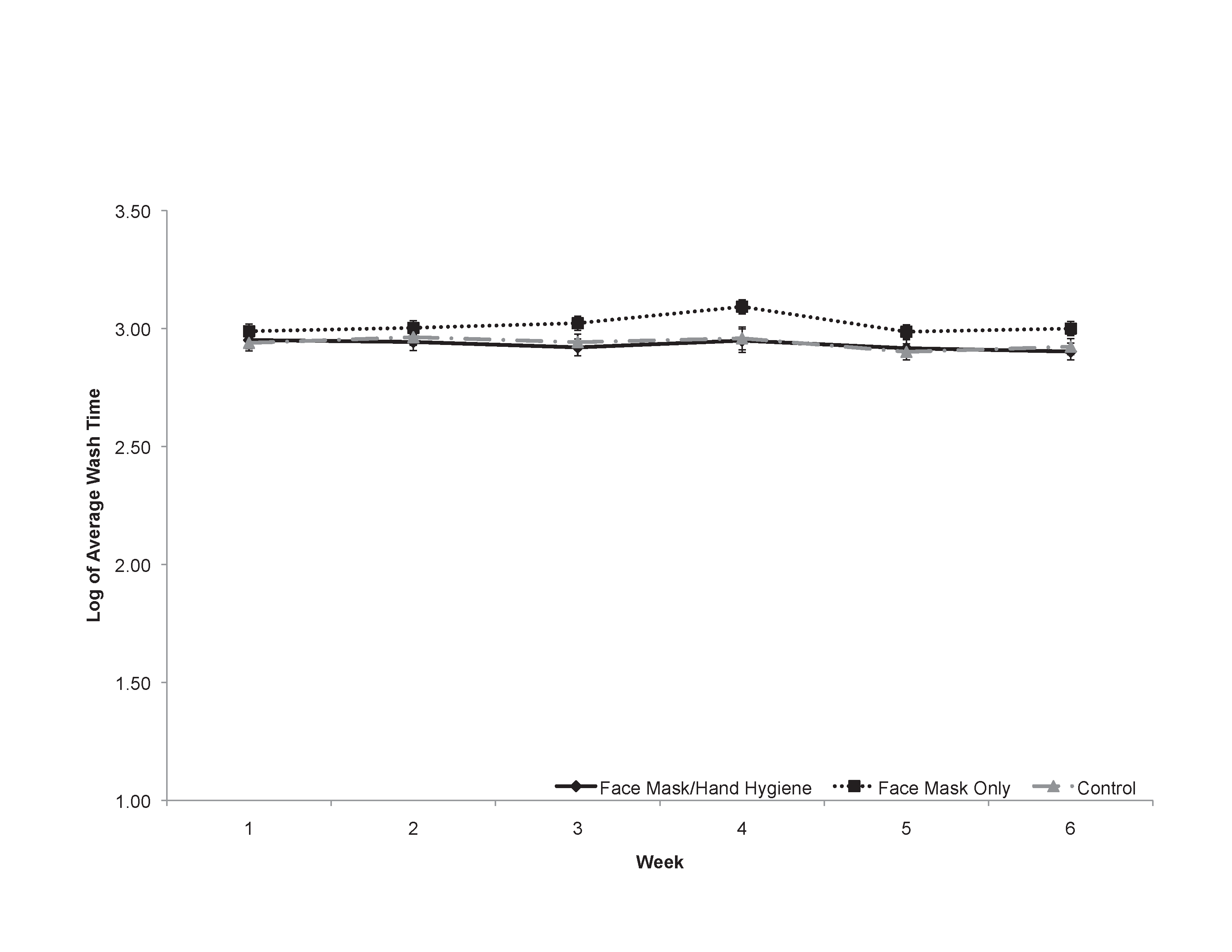

Supplement: Figure S2 — Reported daily average seconds of hand washing (log transformed) by study week. This figure shows the daily average time for washing hands (log transformed) by study week in the face mask and hand hygiene group (solid line), the face mask only group (dotted line), and the control group (dashed line). The type III fixed effects model for assessing differences over time using a week * group interaction term, was not statistically significant, F(10, 4518) = 1.12 and P = 0.34. (TIF) [file pone.0029744.s002.tif]

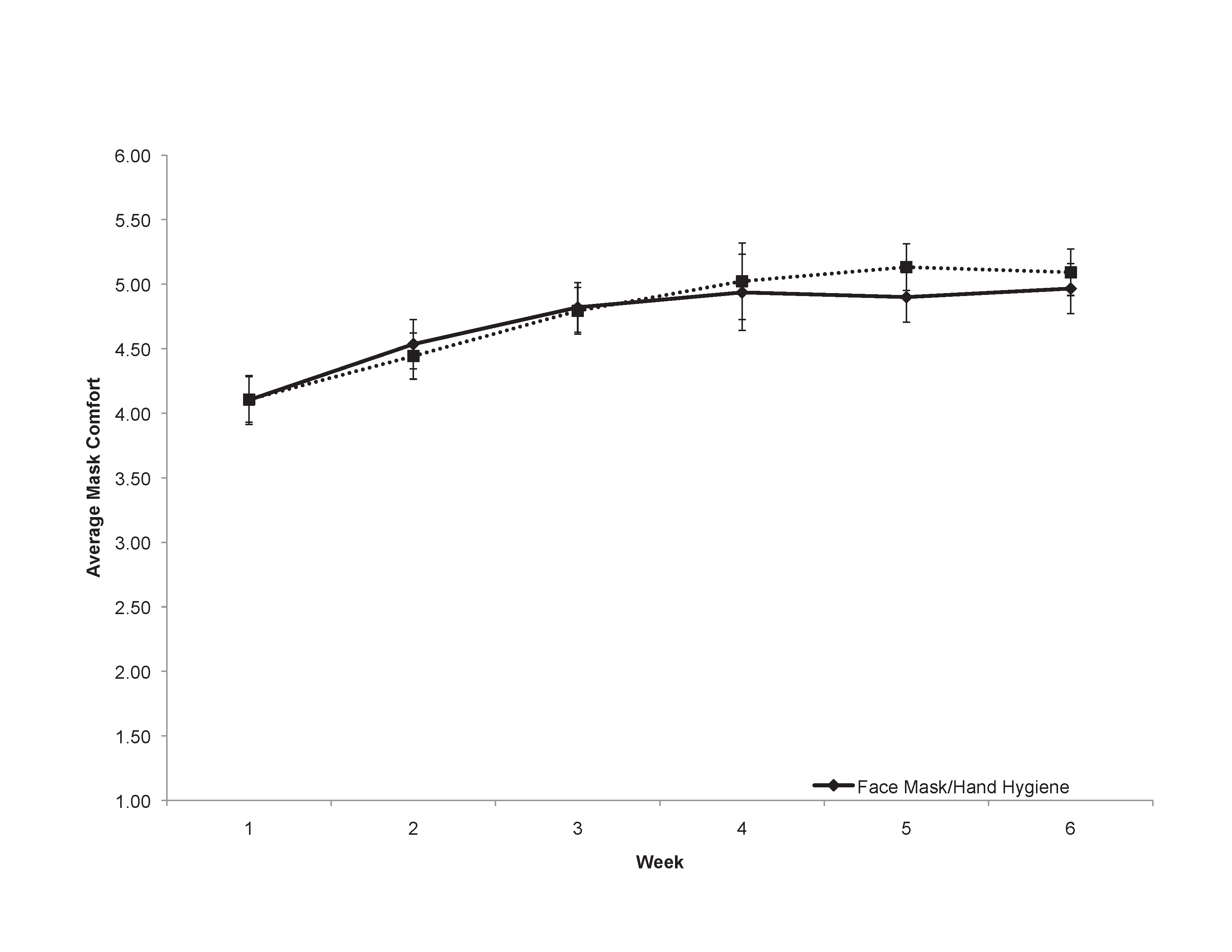

Supplement: Figure S3 — Reported daily average mask comfort rating (log transformed) by study week. This figure shows the daily average mask comfort rating (log transformed) by study week in both the face mask and hand hygiene group (solid line) and the face mask only group (dotted line). The type III fixed effects model for assessing differences over time using a week * group interaction term, was not statistically significant, F(5, 2942) = 0.68 and P = 0.63. (TIF) [file pone.0029744.s003.tif]

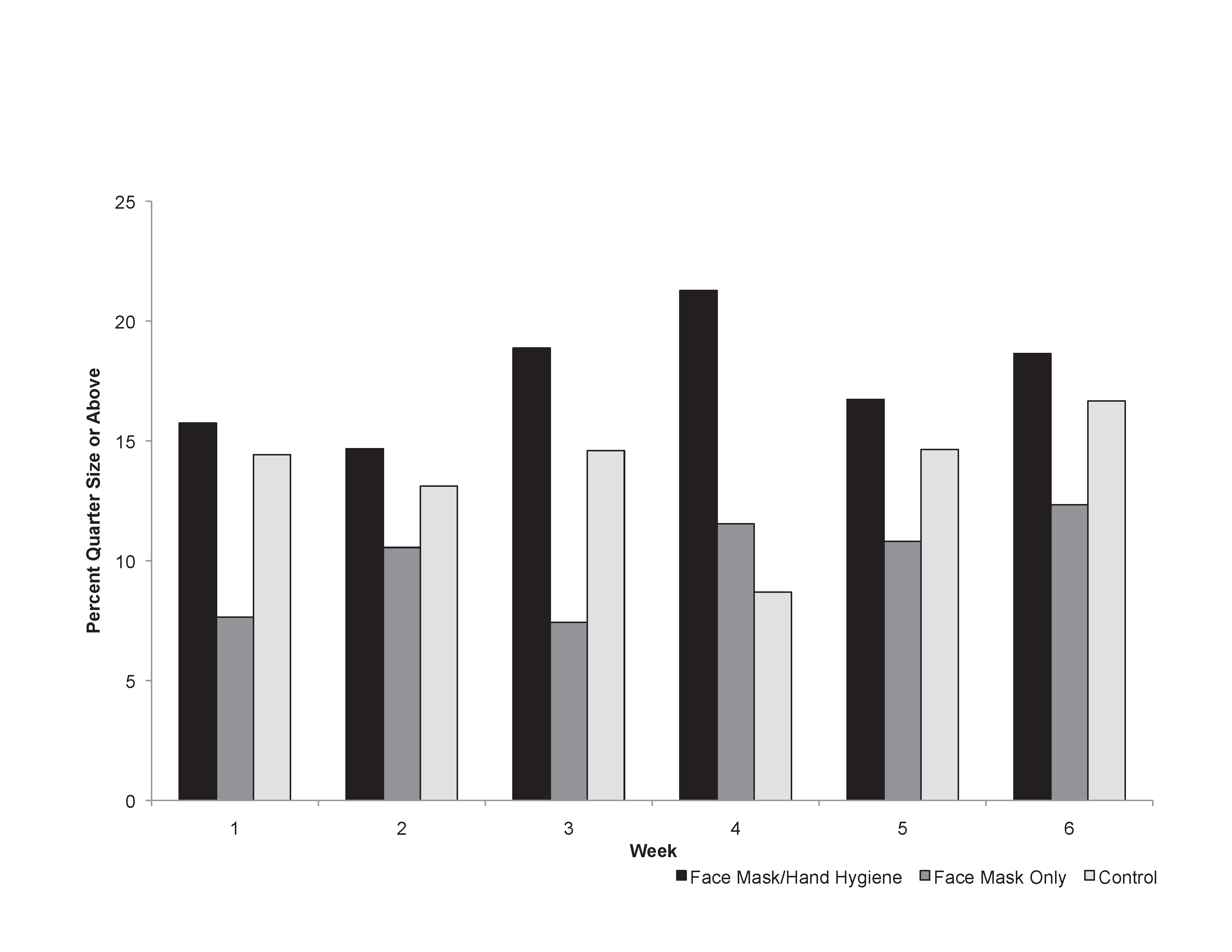

Supplement: Figure S4 — Reported average proportion of proper hand sanitizer use by study week. This figure shows the average proportion of respondents using the proper amount of hand sanitizer (quarter size or larger) when using hand sanitizer by study week in the face mask and hand hygiene group (black), the face mask only group (dark grey), and the control group (light grey). (TIF) [file pone.0029744.s004.tif]

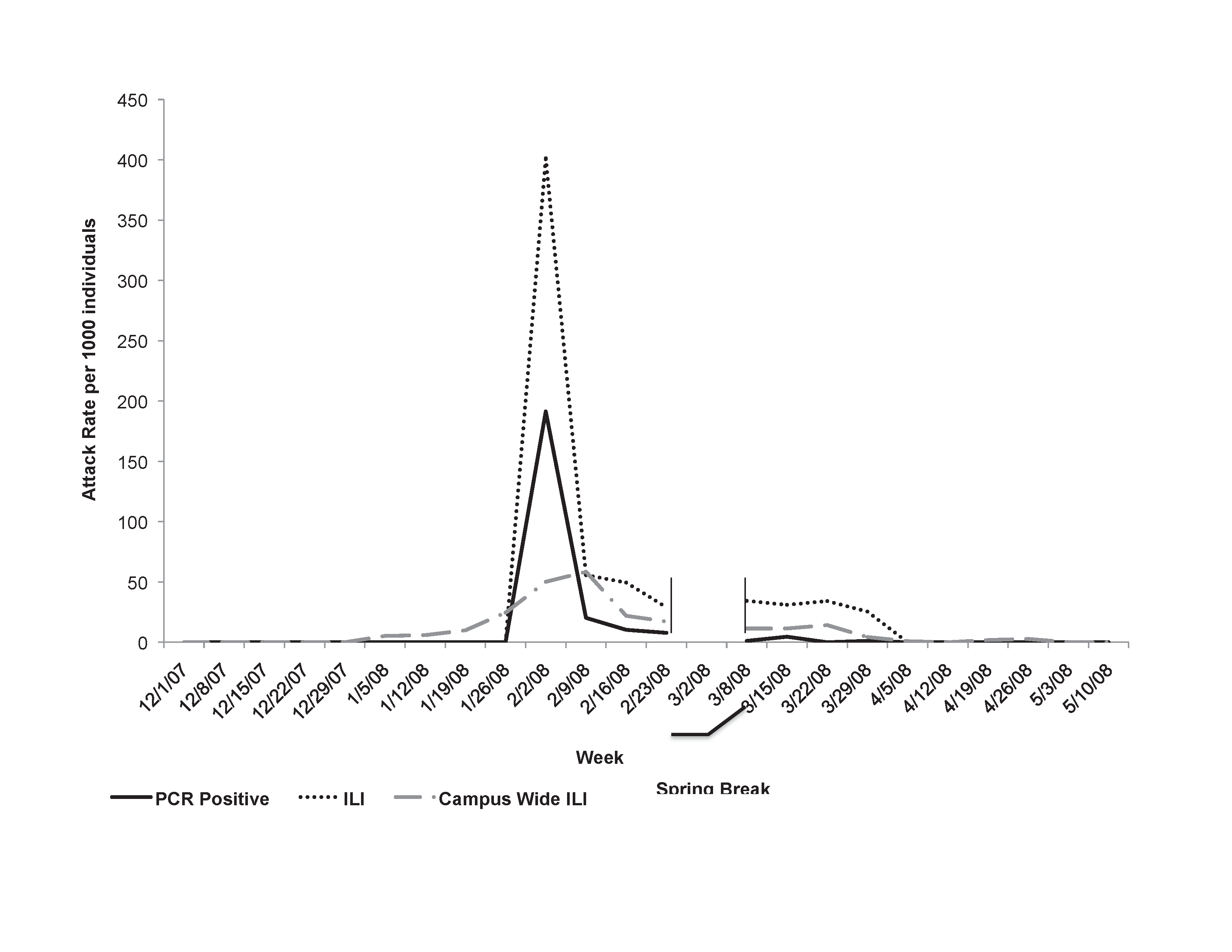

Supplement: Figure S5 — Attack rate of influenza like-illness at the University of Michigan during the 2007–2008 influenza season. The attack rate of influenza and influenza like-illness among respondents and across campus. (TIF) [file pone.0029744.s005.tif]
