# Supplementary material for: Facemasks, Hand Hygiene, and Influenza among Young Adults: A Randomized Intervention Trial
Source: PLoS One. 2012 Jan 25;7(1):e29744. doi: 10.1371/journal.pone.0029744 (PMC3266257; doi:10.1371/journal.pone.0029744)
Supplement: Table S1 — Baseline characteristics of the study population. (DOC) [file pone.0029744.s006.doc]

**Table S1. Baseline characteristics of the study population (N=1,111)^a^**

|  | | | | | | | | | | |
| --- | --- | --- | --- | --- | --- | --- | --- | --- | --- | --- |
|  | **No. (%) of Participants** | | | | | | | | | |
| **Characteristics** | **Overall** | | **ICC^b^** | **Face Mask Hand Hygien** | | **Face Mask Only** | | **Control** | | ***P*^c^** |
| Number of residence houses | 37 | |  | 12 | | 13 | | 12 | |  |
| Average residence house size | 29.9 | |  | 29 | | 30.1 | | 30.8 | |  |
| Total number of participants | 1,111 | |  | 349 | | 392 | | 370 | |  |
|  |  |  |  |  |  |  |  |  |  |  |
| Age in years, mean (SD) ^d^ | 18.95 | (0.9) | 0.11 | 19.01 | (0.9) | 18.95 | (1.0) | 18.90 | (0.9) | 0.99 |
|  |  |  |  |  |  |  |  |  |  |  |
| Gender |  |  | 0.18 |  |  |  |  |  |  | 0.82 |
| Female | 611 | (55) |  | 179 | (52) | 225 | (58) | 207 | (56) |  |
| Male | 496 | (45) |  | 168 | (48) | 166 | (42) | 162 | (44) |  |
|  |  |  |  |  |  |  |  |  |  |  |
| Ethnicity |  |  | 0.02 |  |  |  |  |  |  | 0.93 |
| Hispanic or Latino | 57 | (5) |  | 17 | (5) | 20 | (5) | 20 | (6) |  |
| Not Hispanic or not Latino | 1014 | (95) |  | 316 | (95) | 360 | (95) | 338 | (94) |  |
|  |  |  |  |  |  |  |  |  |  |  |
| Race |  |  | 0.05 |  |  |  |  |  |  | 0.31^e^ |
| White | 690 | (64) |  | 201 | (60) | 261 | (68) | 228 | (64) |  |
| Black/African American | 102 | (9) |  | 37 | (11) | 29 | (8) | 36 | (10) |  |
| Asian | 214 | (20) |  | 70 | (21) | 66 | (17) | 78 | (22) |  |
| Other^f^ | 68 | (6) |  | 25 | (8) | 26 | (7) | 17 | (5) |  |
|  |  |  |  |  |  |  |  |  |  |  |
| Sleep Quality |  |  | 0.007 |  |  |  |  |  |  | 0.94 |
| Very/Fairly Bad | 237 | (22) |  | 76 | (22) | 85 | (22) | 76 | (21) |  |
| Very/Fairly Good | 849 | (78) |  | 265 | (78) | 300 | (78) | 284 | (79) |  |
|  |  |  |  |  |  |  |  |  |  |  |
| Perceived Stress^g^, mean score (SD) | 23.14 | (7.6) | 0.02 | 23.33 | (7.5) | 23.06 | (7.6) | 23.04 | (7.8) | 0.99 |
|  |  |  |  |  |  |  |  |  |  |  |
| Smoking |  |  | 0.02 |  |  |  |  |  |  | 0.19 |
| Current | 30 | (3) |  | 8 | (2) | 15 | (4) | 7 | (2) |  |
| Non-Smoker | 1055 | (97) |  | 334 | (98) | 369 | (96) | 352 | (98) |  |
|  |  |  |  |  |  |  |  |  |  |  |
| Alcohol consumption, drinks/week |  |  | 0.04 |  |  |  |  |  |  | 0.82^h^ |
| 0 | 706 | (66) |  | 221 | (67) | 246 | (65) | 239 | (68) |  |
| 1 to 7 drinks | 174 | (16) |  | 52 | (16) | 62 | (16) | 60 | (17) |  |
| 8 or more drinks | 184 | (18) |  | 59 | (18) | 72 | (19) | 53 | (15) |  |
|  |  |  |  |  |  |  |  |  |  |  |
| Exercise^i^ |  |  | 0 |  |  |  |  |  |  | 0.34 |
| Low Rate | 772 | (72) |  | 241 | (72) | 268 | (70) | 263 | (74) |  |
| High Rate | 300 | (28) |  | 95 | (28) | 114 | (30) | 91 | (26) |  |
|  |  |  |  |  |  |  |  |  |  |  |
| Flu Vaccine |  |  | 0.001 |  |  |  |  |  |  | 0.46 |
| Never | 548 | (52) |  | 180 | (55) | 191 | (51) | 177 | (50) |  |
| Ever | 502 | (48) |  | 147 | (45) | 181 | (49) | 174 | (50) |  |
|  |  |  |  |  |  |  |  |  |  |  |
| Recent Flu Vaccine^j^ |  |  | 0 |  |  |  |  |  |  | 0.53 |
| Yes | 179 | (17) |  | 55 | (16) | 59 | (16) | 65 | (18) |  |
| No | 886 | (83) |  | 279 | (84) | 320 | (84) | 287 | (82) |  |
|  |  |  |  |  |  |  |  |  |  |  |
| Hand Washing^k^ |  |  | 0.02 |  |  |  |  |  |  | 0.67 |
| Optimal | 283 | (26) |  | 95 | (27) | 93 | (24) | 95 | (26) |  |
| Suboptimal | 822 | (74) |  | 251 | (73) | 298 | (76) | 273 | (74) |  |
|  |  |  |  |  |  |  |  |  |  |  |
| Hand Sanitizer Ownership |  |  | 0.03 |  |  |  |  |  |  | 0.54 |
| Yes | 574 | (52) |  | 192 | (56) | 196 | (50) | 186 | (51) |  |
| No | 525 | (48) |  | 152 | (44) | 193 | (50) | 180 | (49) |  |

^a^1,111 participants eligible for intention-to-treat analyses.

^b^ICC=Intracluster Correlation Coefficient, negative ICC values were set to 0.

^c^*P* Values computed using cluster-adjusted chi-square test for categorical characteristics and cluster-adjusted ANOVA for continuous characteristics.

^d^SD, standard deviation; Total of 4 participants missing age, 2 in face mask hand hygiene, 1 in face mask, 1 in control.

^e^Includes Hawaiian, Pacific Islander, American Indian, Alaskan Native, and Multi-Ethnic; 34 participants responded “decline to answer.”

^f^*P* value comparing White vs. all other race groups.

^g^Total of 87 participants missing perceived stress score, 35 in face mask hand hygiene, 22 in face mask, and 30 control.

^h^*P* value comparing zero drinks per week vs. one or more.

^i^High rate defined as exercising at a very or extremely hard rate for at least 20 minutes, 3 or more times per week or exercising at an easy, medium, or hard rate for at least 30 minutes, 5 or more times per week.

^j^Flu vaccination for the 2007-2008 flu season was measured at baseline. Respondents categorized as having recently been vaccinated according to whether they said “yes” to recent vaccination on either survey.

^k^Optimal hand washing defined as washing 5 or more times per day and for at least 20 seconds.
