# Supplementary material for: Facemasks, Hand Hygiene, and Influenza among Young Adults: A Randomized Intervention Trial
Source: PLoS One. 2012 Jan 25;7(1):e29744. doi: 10.1371/journal.pone.0029744 (PMC3266257; doi:10.1371/journal.pone.0029744)
Supplement: Table S2 — Log reported average daily hand washing per week and P values comparing average washing in each group with face mask and hand hygiene. (DOC) [file pone.0029744.s007.doc]

**Table S2. Log reported average daily hand washing per week and *P* values comparing average washing in each group with face mask and hand hygiene**

| **Intervention** | | **Average over all weeks^a^** | **Week 1** | **Week 2** | **Week 3** | **Week 4** | **Week 5** | **Week 6** |
| --- | --- | --- | --- | --- | --- | --- | --- | --- |
| Face Mask and Hand hygiene | | 1.72 | 1.74 | 1.73 | 1.73 | 1.68 | 1.71 | 1.71 |
|  | vs. Face Mask Only^b^ | 1.76 | 1.74 | 1.74 | 1.77 | 1.74 | 1.75 | 1.79 |
|  |  |  | (*P* = 0.91) | (*P* = 0.81) | (*P* = 0.40) | (*P* = 0.39) | (*P* = 0.36) | (*P* = 0.10) |
|  | vs. Control | 1.78 | 1.74 | 1.77 | 1.78 | 1.74 | 1.77 | 1.78 |
|  |  |  | (*P* = 0.97) | (*P* = 0.28) | (*P* = 0.30) | (*P* = 0.36) | (*P* = 0.16) | (*P* = 0.15) |

^a^The change in reported average log transformed daily hand washing over the 6 week period comparing between all three study groups (week by group interaction term) using a Type III fixed effects model resulted in an *F*(10, 4543)=1.43 and *P* = 0.16.

^b^There were no statistically significant differences at any weeks comparing daily hand washing between face mask only and the control group (all *P* > 0.025).
