# Supplementary material for: Facemasks, Hand Hygiene, and Influenza among Young Adults: A Randomized Intervention Trial
Source: PLoS One. 2012 Jan 25;7(1):e29744. doi: 10.1371/journal.pone.0029744 (PMC3266257; doi:10.1371/journal.pone.0029744)
Supplement: Table S3 — Log reported average wash time in seconds per week and P values comparing wash time in each group with face mask and hand hygiene. (DOC) [file pone.0029744.s008.doc]

**Table S3. Log reported average wash time in seconds per week and *P* values comparing wash time in each group with face mask and hand hygiene**

| **Intervention** | | **Average over all weeks^a^** | **Week 1** | **Week 2** | **Week 3** | **Week 4** | **Week 5** | **Week 6** |
| --- | --- | --- | --- | --- | --- | --- | --- | --- |
| Face Mask and Hand Hygiene | | 2.92 | 2.95 | 2.94 | 2.92 | 2.95 | 2.92 | 2.90 |
|  | vs. Face Mask Only^b^ | 3.01 | 2.99 | 3.00 | 3.02 | 3.09 | 2.99 | 3.00 |
|  |  |  | (*P* = 0.44) | (*P* = 0.22) | (*P* = 0.04) | (*P* = 0.04) | (*P* = 0.16) | (*P* = 0.05) |
|  | vs. Control | 2.92 | 2.94 | 2.96 | 2.94 | 2.96 | 2.90 | 2.92 |
|  |  |  | (*P* = 0.81) | (*P* = 0.68) | (*P* = 0.66) | (*P* = 0.89) | (*P* = 0.76) | (*P* = 0.67) |

^a^The change in reported average log transformed wash time in seconds over the 6 week period comparing between all three study groups (week by group interaction term) using a Type III fixed effects model resulted in an *F*(10, 4518)=1.12 and *P* = 0.34.

^b^There were no statistically significant differences at any weeks comparing daily hand washing between face mask only and the control group (all *P* > 0.025).
