# Supplementary material for: Facemasks, Hand Hygiene, and Influenza among Young Adults: A Randomized Intervention Trial
Source: PLoS One. 2012 Jan 25;7(1):e29744. doi: 10.1371/journal.pone.0029744 (PMC3266257; doi:10.1371/journal.pone.0029744)
Supplement: Table S4 — Log reported average face mask comfort per week and P values comparing comfort in the face mask only group with face mask and hand hygiene. (DOC) [file pone.0029744.s009.doc]

**Table S4. Log reported average face mask comfort per week and *P* values comparing comfort in the face mask only group with face mask and hand hygiene**

| **Intervention** | | **Average over all weeks^a^** | **Week 1** | **Week 2** | **Week 3** | **Week 4** | **Week 5** | **Week 6** |
| --- | --- | --- | --- | --- | --- | --- | --- | --- |
| Face Mask Hand Hygiene | | 4.71 | 4.10 | 4.54 | 4.82 | 4.94 | 4.90 | 4.97 |
|  | vs. Face Mask Only | 4.77 | 4.11 | 4.44 | 4.79 | 5.02 | 5.13 | 5.09 |
|  |  |  | (*P* = 0.99) | (*P* = 0.72) | (*P* = 0.92) | (*P* = 0.84) | (*P* = 0.38) | (*P* = 0.63) |

^a^The change in reported average log transformed face mask comfort over the 6 week period comparing between the two intervention groups (week by group interaction term) using a Type III fixed effects model resulted in an F(5, 2942)=0.68 and P = 0.63.
