# Supplementary material for: Facemasks, Hand Hygiene, and Influenza among Young Adults: A Randomized Intervention Trial
Source: PLoS One. 2012 Jan 25;7(1):e29744. doi: 10.1371/journal.pone.0029744 (PMC3266257; doi:10.1371/journal.pone.0029744)
Supplement: Table S5 — Proportion of influenza-like illness cases who tested positive for influenza infection as determined by polymerase chain reaction (PCR). (DOC) [file pone.0029744.s010.doc]

**Table S5. Proportion of influenza-like illness cases who tested positive for influenza infection as determined by polymerase chain reaction (PCR)a**

| **Characteristics** | **Overall, n (%)** | | **Face Mask/Hand Hygiene** | | **Face Mask Only** | | **Control** | |
| --- | --- | --- | --- | --- | --- | --- | --- | --- |
| Total number of ILI cases | 128 | | 31 | | 46 | | 51 | |
|  |  |  |  |  |  |  |  |  |
| PCR |  |  |  |  |  |  |  |  |
| Positive | 34 | (27) | 6 | (19) | 12 | (26) | 16 | (31) |
| Negative | 94 | (73) | 25 | (81) | 34 | (74) | 35 | (69) |
|  |  |  |  |  |  |  |  |  |

^a^PCR used to detect influenza A and B viruses.
