# Supplementary material for: Facemasks, Hand Hygiene, and Influenza among Young Adults: A Randomized Intervention Trial
Source: PLoS One. 2012 Jan 25;7(1):e29744. doi: 10.1371/journal.pone.0029744 (PMC3266257; doi:10.1371/journal.pone.0029744)
Supplement: Table S6 — Proportion of subjects using a quarter or greater amount of alcohol sanitizer and P values comparing each group using the Donner and Donald chi-square test. (DOC) [file pone.0029744.s011.doc]

**Table S6. Proportion of subjects using a quarter or greater amount of alcohol sanitizer and *P* values comparing each group using the Donner and Donald chi-square test^a^**

| **Intervention** | **Average over all weeks** | **Week 1** | **Week 2** | **Week 3** | **Week 4** | **Week 5** | **Week 6** |
| --- | --- | --- | --- | --- | --- | --- | --- |
| Face Mask and Hand Hygiene | 0.17 | 0.16 | 0.15 | 0.19 | 0.21 | 0.17 | 0.19 |
| Face Mask Only | 0.10 | 0.08 | 0.11 | 0.07 | 0.12 | 0.11 | 0.12 |
| Control | 0.15 | 0.14 | 0.13 | 0.15 | 0.09 | 0.15 | 0.17 |
| Adjusted χ^2^ |  | *P =* 0.13 | *P =* 0.63 | *P =* 0.04 | *P =* 0.16 | *P =* 0.36 | *P =* 0.49 |

^a^P Values were calculated using the Donner and Donald chi-square test [7].
