# Supplementary material for: Facemasks, Hand Hygiene, and Influenza among Young Adults: A Randomized Intervention Trial
Source: PLoS One. 2012 Jan 25;7(1):e29744. doi: 10.1371/journal.pone.0029744 (PMC3266257; doi:10.1371/journal.pone.0029744)
Supplement: Table S7 — Observational data based on the total hours of observation in each residence hall and the percentage of shifts in which participants were seen properly wearing facemasks. (DOC) [file pone.0029744.s012.doc]

| **Table S7. Observational data based on the total hours of observation in each residence hall and the percentage of shifts in which participants were seen properly wearing facemasks** | | | | | | | | | | |
| --- | --- | --- | --- | --- | --- | --- | --- | --- | --- | --- |
|  | Alice Lloyd | | Bursley | | East Quad | | South Quad | | West Quad | |
|  | % of shifts^a^ | Total Hours Observed | % of shifts^a^ | Total Hours Observed | % of shifts^a^ | Total Hours Observed | % of shifts^a^ | Total Hours Observed | % of shifts^a^ | Total Hours Observed |
| Week 1 | 41.2 | 16.5 | 0.0 | 29.1 | 41.7 | 23.2 | 21.4 | 27.2 | 19.2 | 27.9 |
| Week 2 | 0.0 | 10.8 | 30.0 | 32.8 | 27.3 | 20.8 | 6.1 | 32.5 | 4.2 | 24.3 |
| Week 3 | 4.8 | 20.1 | 32.4 | 42.3 | 12.2 | 39.5 | 15.2 | 45.0 | 4.9 | 40.3 |
| Week 4 | 0.0 | 10.5 | 23.3 | 26.6 | 0.0 | 26.9 | 0.0 | 31.3 | 2.9 | 33.0 |
| Week 5 | 10.0 | 19.4 | 14.8 | 46.0 | 12.5 | 38.6 | 6.0 | 48.1 | 0.0 | 41.2 |
| Week 6 | 0.0 | 18.1 | 19.6 | 43.8 | 0.0 | 41.6 | 1.9 | 50.8 | 4.1 | 48.5 |
| ^a^Percentage of shifts that participants were seen properly wearing provided face masks by staff observations. | | | | | | | | | | |
