# Supplementary material for: Facemasks, Hand Hygiene, and Influenza among Young Adults: A Randomized Intervention Trial
Source: PLoS One. 2012 Jan 25;7(1):e29744. doi: 10.1371/journal.pone.0029744 (PMC3266257; doi:10.1371/journal.pone.0029744)
Supplement: Text S1 — Additional methods and compliance measures. This text provides additional information about randomization and distribution of supplies, behavioral measures collected, additional compliance measures, laboratory methods and study attack rate. (DOC) [file pone.0029744.s013.doc]

**Supporting Information**

I. Randomization Procedures and Distribution of Supplies. Randomization was conducted at the residence house level (n=37). Residence houses were contained within each of the five residence halls in the study (n = 5), thus ensuring that each residence hall had residence houses assigned to each intervention and control arm. Each sequence (i.e. ordering) of the intervention and control arms was assigned a value. For example, sequence (i.e. order) 1 consisted of face mask and hand hygiene; face mask only; and control. Sequence (i.e. order) 2 consisted of face mask and hand hygiene; control; face mask only, and so on. Then, each sequence was randomly selected, whereby each had an equal probability of being selected. The first residence house within the first residence hall was then selected and assigned the first value for the randomized sequence (for sequence 1, house 1 would be assigned face mask and hand hygiene), followed by the second house being assigned the sequence (sequence 1, face mask only), and the third house being assigned the final treatment in the sequence (sequence 1, control). In each residence hall, as there was greater than three houses, more than one sequence was selected and each house was drawn until all were assigned an intervention. This process ensured that each residence house had an equal probability of being assigned to the interventions or control arm and also that each residence hall would have at least one of each intervention and control groups.

The majority of participants included in the analysis (933/1,111, 84%) filled out a baseline survey and reported on baseline ILI prior to the intervention start. The proportion of participants that were allowed to enroll between January 28th to February 12th was similar across intervention (face mask only= 16%, face mask and hand hygiene = 15%) and control (17%) groups. The intervention sequence was concealed prior to the start of the intervention period.

Participants in the face mask and hand hygiene and the face mask only groups received weekly packets of mask supplies in their student mailboxes. Student signature of a packet slip confirmed receipt of mask packets. All students in the face mask and hand hygiene intervention also received hand sanitizer (2 oz squeeze bottle, 8 oz pump bottle) labeled with their study identification number. Study-staffed tables located in each residence hall offered a surplus of face masks and hand sanitizer (with exchange of used, empty bottles) to ensure a supply chain of intervention materials. As with mask packets, participants’ signatures were logged to confirm receipt of surplus supplies.

II. Behavioral Measures. Questions related to hand hygiene were asked of all study participants. A variable for optimal hand washing was constructed using the CDC recommendations of washing for at least 20 seconds with soap and water and an average of five or more times per day, as reported by study subjects. Participants whose average hand washing behavior fell below 20 seconds or less than five times per day were considered to have “sub-optimal” hand hygiene and subjects reporting average values at or above 20 seconds and five times per day were considered to have “optimal” hand hygiene. Sleep quality was recorded on a 4-point scale based on quality of sleep in the previous month. Participants were categorized according to “fairly/very good sleep” and “fairly/very bad sleep”. Tobacco use (yes/no) and alcohol consumption (0-1 drinks versus 2 or more drinks per week) were determined at baseline. Participants were coded as having “high” physical activity at baseline if they reported exercising hard or extremely hard for 20 or more minutes at least 3 times per week or at any exertion for at least 30 minutes 5 or more times per week. All other subjects were classified as engaging in “low” physical activity. Perceived stress was measured at baseline using a 14-item validated scale [1] with excellent internal validity.

III. Additional Compliance Measures and Analyses. In addition to compliance with the intervention materials (mask use hours and hand sanitizer frequency) described in the main text, additional compliance measures collected on weekly surveys including number of hand washes per day, duration of hand washing in seconds, levels of comfort with mask wearing, and amount of hand sanitizer used are presented here. Among participants in the facemask only and control groups, the use of personally supplied alcohol-based hand sanitizer was examined. These study groups were not provided study-associated hand sanitizer.

Compliance assessments for reported average number of hand washes and average duration of hand washing in seconds were log transformed to normalize skewed data. Mask comfort, measured from 0 being uncomfortable and 10 being comfortable, was not transformed since the distribution was normal. In order to prevent data loss due to the transformation, a value of one was added to all variables before being log-transformed. Differences between intervention and control groups in compliance were examined using a multi-level mixed model that accounted for clustering at the residence house level. Level-1 of the model accounted for changes between individuals over the course of the study period. The second level allowed for changes within individuals across the study period. Random intercepts were allowed to account for clustering at the residence house level. We adjusted the *P* value required for significance to 0.025 to account for multiple comparisons over the study period.

When comparing hand washing behavior between study groups, the facemask and hand hygiene group washed their hands 5.20 times per day (SD, 3.33) versus 5.49 times per day (SD, 3.30) in the mask only group during the study. Subjects in the control group washed their hands an average of 5.81 times per day (SD, 5.03). On the log scale, mixed model analyses showed no statistical differences between any of the groups (*F*(10, 4543)=1.43 and *P* = 0.16 ) (Table S2 and Figure S1).

Subjects in the facemask and hand hygiene group washed their hands an average of 20.53 seconds per day (SD, 12.21) compared to 22.36 seconds per day (SD, 13.07) among subjects in the facemask only group. Participants in the control group washed their hands on average 20.56 seconds per day (SD, 12.68). On the log scale, the mask and hand hygiene group reported a significantly shorter duration of hand washing at weeks 3, 4 and 6 compared to the facemask only group (Table S3 and Figure S2).

On average, the face mask and hand hygiene group rated mask comfort as a 4.71 (SD, 0.21) out of 10 (comfortable) compared to 4.77 (SD, 0.20) out of 10 for the facemask only group. There were no significant differences in mask comfort between interventions at each time period, but both groups showed a gradual increase in comfort level throughout the study period (Table S4 and Figure S3).

Amount of hand sanitizer use was dichotomized into equal to or greater than the size of a quarter compared to less than a quarter size amount. A cluster adjusted chi-square test [2] was performed to examine differences in the amount of hand sanitizer used between study groups. During week 4 of the study, there was a significantly higher proportion of participants in the mask and hand hygiene intervention group using a quarter size or greater amount of alcohol-based hand sanitizer compared to the mask only and control groups (*P=*0.04). There were no other significant differences at other time points between study groups (Table S6 and Figure S4).

Observed Compliance. Mask compliance was also examined within residence halls via observational data recorded by trained study staff. Staff anonymously observed the number of individuals wearing masks, both correctly and incorrectly, in public areas throughout the residence halls on a daily basis. No contact was made between study staff and students. A total of 1308 hours of observation were collected. Observational data were analyzed based on the total hours of observation in each residence hall and the percentage of shifts in which participants were seen properly wearing facemasks. Staff observed an average of 0.0007 participants properly wearing a mask for each hour of observation over the six week study period (see Table S7).

IV. Laboratory methods. Requirements for providing a throat swab sample from study participants were based on survey reported symptoms of cough plus at least one or more of fever/feverishness, body aches, or chills. The primers used for our analysis were synthesized commercially by IDT DNA (Coralville, IA) and Biosearch Technologies (Novato, CA). Additional information, including probes, cycling conditions and primers is available upon request from the authors. Following testing, samples were stored at -70 degrees Celsius.

V. Study and campus attack rates per 1,000 individuals. The PCR positive attack rate in our study was defined by the number of positive PCR samples gathered per total number of study subjects responding each week. The peak reflects the high proportion of PCR confirmed cases early in our study (see Figure S5). The ILI attack rate is defined by the number of ILI reports per total number of study subjects responding each week. Campus-wide ILI is defined by the number of weekly reported ILI cases provided by University Health Services per total number of patients seen at the University Health Services (see Figure S5). The campus-wide ILI rate reflects a much larger population than our study population. It additionally does not capture students who sought treatment outside of the University Health Service system. The spring break one-week period ending March 1, 2008 was excluded in our calculations (see Figure S5).

References:

1. Cohen S, Kamarck T, Mermelstein R (1983) A global measure of perceived stress. J Health Soc Behav 24: 385-396.

2. Donner A, Donald A (1988) The statistical analysis of multiple binary measurements. J Clin Epidemiol 41: 899-905.
