# Supplementary material for: Facemasks, Hand Hygiene, and Influenza among Young Adults: A Randomized Intervention Trial
Source: PLoS One. 2012 Jan 25;7(1):e29744. doi: 10.1371/journal.pone.0029744 (PMC3266257; doi:10.1371/journal.pone.0029744)
Supplement: Checklist S1 — CONSORT checklist. This text provides the clustered CONSORT checklist for our study. (DOC) [file pone.0029744.s014.doc]

CONSORT Checklist

| ***PAPER SECTION* And topic** | **Item** | **Descriptor** | **Reported on Page #** |
| --- | --- | --- | --- |
| ***TITLE & ABSTRACT*** |  |  |  |
| *Design* | 1 | How participants were allocated to interventions (*e.g*., "random allocation", "randomized", or "randomly assigned"), *specifying that allocation was based on clusters* | Introduction |
| ***INTRODUCTION*** |  |  |  |
| Background | 2 | Scientific background and explanation of rationale, *including the rationale for using a cluster design* | Introduction |
| ***METHODS*** |  |  |  |
| Participants | 3 | Eligibility criteria for participants and clusters and the settings and locations where the data were collected | Methods |
| Interventions | 4 | Precise details of the interventions intended for each group, *whether they pertain to the individual level, the cluster level, or both,* and how and when they were actually administered | Methods |
| Objectives | 5 | Specific objectives and hypotheses *and whether they pertain to the individual level, the cluster level, or both* | Methods |
| Outcomes | 6 | Clearly defined primary and secondary outcome measures *whether they pertain to the individual level, the cluster level, or both*, and, when applicable, any methods used to enhance the quality of measurements (*e.g.*, multiple observations, training of assessors) | Methods |
| Sample size | 7 | How *total* sample size was determined *(including method of calculation, number of clusters, cluster size, a coefficient of intracluster correlation (ICC or k), and an indication of its uncertainty*) and, when applicable, explanation of any interim analyses and stopping rules | Methods |
| *Randomization* |  |  |  |
| Sequence generation | 8 | Method used to generate the random allocation sequence, including details of any restrictions (*e.g*., blocking, stratification, matching) | Methods, Text S1 |
| Allocation concealment | 9 | Method used to implement the random allocation sequence, *specifying that allocation was based on clusters rather than individuals and* clarifying whether the sequence was concealed until interventions were assigned | Methods, Text S1 |
| Implementation | 10 | Who generated the allocation sequence, who enrolled participants, and who assigned participants to their groups | Methods |
| Blinding (masking) | 11 | Whether or not participants, those administering the interventions, and those assessing the outcomes were blinded to group assignment. If done, how the success of blinding was evaluated | Methods |
| Statistical methods | 12 | Statistical methods used to compare groups for primary outcome(s) *indicating how clustering was taken into account*; methods for additional analyses, such as subgroup analyses and adjusted analyses | Methods |
| ***RESULTS*** |  |  |  |
| Participant flow | 13 | Flow of clusters and participants through each stage (a diagram is strongly recommended). Specifically, for each group report the numbers of clusters and participants randomly assigned, receiving intended treatment, completing the study protocol, and analyzed for the primary outcome. Describe protocol deviations from study as planned, together with reasons. | Results, Figure 1 |
| Recruitment | 14 | Dates defining the periods of recruitment and follow-up | Methods, Figure 1 |
| Baseline data | 15 | Baseline demographic and clinical characteristics of each group *for the individual and cluster levels as applicable* | Results, Tables 1-2, Table S1 |
| Numbers analyzed | 16 | Number of clusters and participants (denominator) in each group included in each analysis and whether the analysis was by "intention-to-treat". State the results in absolute numbers when feasible (*e.g*., 10/20, not 50%). | Results, Tables 1-4, Table S1 |
| Outcomes and estimation | 17 | For each primary and secondary outcome, a summary of results for each group for the individual or cluster as applicable, and the estimated effect size and its precision (*e.g.*, 95% confidence interval) | Results, Tables 3-4 |
| Ancillary analyses | 18 | Address multiplicity by reporting any other analyses performed, including subgroup analyses and adjusted analyses, indicating those pre-specified and those exploratory | Results, Table 1, Text S1 |
| Adverse events | 19 | All important adverse events or side effects in each intervention group | NA |
| ***DISCUSSION*** |  |  |  |
| Interpretation | 20 | Interpretation of the results, taking into account study hypotheses, sources of potential bias or imprecision and the dangers associated with multiplicity of analyses and outcomes | Discussion |
| Generalizability | 21 | Generalizability (external validity) *to individuals and/or clusters (as relevant)* of the trial findings | Discussion |
| Overall evidence | 22 | General interpretation of the results in the context of current evidence | Discussion |
