# Supplementary material for: Facemasks, Hand Hygiene, and Influenza among Young Adults: A Randomized Intervention Trial
Source: PLoS One. 2012 Jan 25;7(1):e29744. doi: 10.1371/journal.pone.0029744 (PMC3266257; doi:10.1371/journal.pone.0029744)
Supplement: Protocol S1 — Trial protocol. This text provides additional information about the protocol of the study. (PDF) [file pone.0029744.s015.pdf]

University of Michigan School of Public Health
Reducing Transmission of Influenza by Face Masks (M-FLU)

RECRUITMENT

We sought to recruit approximately 2,200 subjects and final recruitment numbers resulted in a total of 1,178 subjects.

RECRUITMENT MATERIALS

All materials are IRB approved before dissemination.

Recruitment materials such as highlighters, posters, table tents and flyers to advertise the study to students in the five residence were developed by a graphics professional. The same images were used to develop the website http://www.sph.umich.edu/mflu/ so that recruitment and enrollment were synchronized into one effort.

RECRUITMENT METHODS

Recruitment efforts are designed to comply with IRB approved methods. Research subjects are never induced to participate by means other than voluntary consent and with knowledge of the research study and its requirements. Recruiting staff are taught to avoid coercion or undue influence when recruiting and enrolling potential participants.

Participant recruitment methods included information tables in the residence halls and at festivals, sponsored television and movie nights within the residence halls, chalking on the sidewalks outside of the residence halls, and other residence hall events. Students attending sponsored events were eligible for raffled items, including sweatshirts, t-shirts, board games, snacks. Students enrolled in the study were eligible for weekly drawings for iPod mini music players. Study staff were paired with resident staff for active recruitment during dining hours, events and for the distribution of materials and supplies. Additionally, recruitment materials were placed in all students school mailboxes to provide information about the study and the recruitment process.

ENROLLMENT

The M-FLU study was open to enrollment of all participants in eligible residence halls. In year two, participants eligible for enrollment were contacted either in person or by email and given instructions for web enrollment. Potential participants were able to access enrollment forms from any computer with internet access. Using an in-house survey system, participants were supplied with a consent form, eligibility form, demographic survey, and weekly surveys containing intervention and illness information. If the participants had questions or concerns they were directed to contact the Study Coordinator either via e-mail or telephone.

ELIGIBILITY

Year two of the intervention study only required that the participants live within one of the chosen residence halls (intervention and control) and are at least 18 years of age. In addition participants with an allergy to alcohol-based hand sanitizer were asked to exclude themselves from the study. Participants must, however, be willing to wear a face mask and/or use hand hygiene on a weekly basis, and to have a throat swab specimen collected if they have signs of illness during the influenza season.

ASSIGNMENT OF STUDY ID

Participants who are eligible for participation are assigned a unique Study ID number. Participant information including but not limited to name, address, date of birth and residence hall are entered into the electronic survey system along with their corresponding Study ID.
